# Exogenous 3-Iodothyronamine (T_1_AM) Can Affect Phosphorylation of Proteins Involved on Signal Transduction Pathways in In Vitro Models of Brain Cell Lines, but These Effects Are Not Strengthened by Its Catabolite, 3-Iodothyroacetic Acid (TA_1_)

**DOI:** 10.3390/life12091352

**Published:** 2022-08-30

**Authors:** Lavinia Bandini, Ginevra Sacripanti, Marco Borsò, Maria Tartaria, Maria Pia Fogliaro, Giulia Giannini, Vittoria Carnicelli, Matteo Emanuele Figuccia, Sara Verlotta, Fiammetta De Antoni, Riccardo Zucchi, Sandra Ghelardoni

**Affiliations:** Department of Pathology, University of Pisa, 56126 Pisa, Italy

**Keywords:** 3-iodothyronamine, 3-iodothyroacetic acid, brain, glutamatergic system, protein phosphorylation

## Abstract

T_1_AM, a derivative of thyroid hormones, and its major catabolite, TA_1_, produce effects on memory acquisition in rodents. In the present study, we compared the effects of exogenous T_1_AM and TA_1_ on protein belonging to signal transduction pathways, assuming that TA_1_ may strengthen T_1_AM’s effects in brain tissue. A hybrid line of cancer cells of mouse neuroblastoma and rat glioma (NG 108-15), as well as a human glioblastoma cell line (U-87 MG) were used. We first characterized the in vitro model by analyzing gene expression of proteins involved in the glutamatergic cascade and cellular uptake of T_1_AM and TA_1_. Then, cell viability, glucose consumption, and protein expression were assessed. Both cell lines expressed receptors implicated in glutamatergic pathway, namely Nmdar1, Glur2, and EphB2, but only U-87 MG cells expressed TAAR1. At pharmacological concentrations, T_1_AM was taken up and catabolized to TA_1_ and resulted in more cytotoxicity compared to TA_1_. The major effect, highlighted in both cell lines, albeit on different proteins involved in the glutamatergic signaling, was an increase in phosphorylation, exerted by T_1_AM but not reproduced by TA_1_. These findings indicate that, in our in vitro models, T_1_AM can affect proteins involved in the glutamatergic and other signaling pathways, but these effects are not strengthened by TA_1_.

## 1. Introduction

Thyronamines represent a new class of endogenous signaling compounds detected in blood and tissues of humans and of several animals, which have been postulated to act as neurotransmitters [1,2,3]. Among thyronamines, 3-iodothyronamine (T_1_AM) has been the compound with more physiological effects [1,2]. Probably derived from decarboxylation and deiodination of thyroid hormone thyroxine (T4) [4], it is catabolized to 3-iodothyroacetic acid (TA_1_) by oxidative deamination [5].

T_1_AM can activate G protein coupled receptors, such as Trace Amine Associated Receptor (TAAR)1 [1], even though interactions with other targets, such as plasma membrane transporters or vesicular biogenic amine transporters, cannot be excluded [6,7]. Significant functional effects have also been observed in the central nervous system: regarding its similarity with monoaminergic neurotransmitters, T_1_AM can inhibit the transport of catecholamines, either vesicular or through the plasma membrane, discovering a novel role for T_1_AM as a neuromodulator [6]. Several findings have led to consider T_1_AM as an endogenous adrenergic-blocking neuromodulator at level of the central noradrenergic system [8].

In 2013, Manni et al. [9] observed an enhancement of learning and memory after intracerebral ventricle injections of T_1_AM in mice. The behavioral effects of T_1_AM may be achieved through the modulation of intracellular pathways, counteracting cell stress signaling and leading to the increase in ERK 1/2 phosphorylation and cFos expression [9,10], which have been demonstrated to play a fundamental role in plasticity mechanisms and in memory processes [11,12].

Interestingly, many of the effects attributed to T_1_AM or to its major metabolite TA_1_ appear to oppose those of the classical thyromimetic effects exerted by T3 [13] as a potent hypothermia, a decrease in heart rate and cardiac output [1], and the induction of a shift from carbohydrates to fatty acids as preferential metabolic source [14]. Therefore, some investigators consider T_1_AM as a multitarget ligand, but the patho-physiological role of the individual receptors and binding sites is presently under debate. In general, both T_1_AM and TA_1_ targets seem to overlap. TA_1_ can be virtually produced in every tissue, considering the wide distribution of monoamine oxidases [15]. At doses close to the endogenous levels, TA_1_ modified behavior, favored memory acquisition and hyperglycaemia, and reduced nociceptive thresholds via histamine H1 and H2 receptors [16]. These results led to the speculation that effects of T_1_AM may be due, at least in part, to TA_1_ production.

Glutamatergic neurotransmission, the major excitatory system in the brain, plays a key role in regulating neuroplasticity, neural development, learning, and memory, and it is often compromised in neurological disorders [17]. These processes involve the recruitment of multiple signaling pathways and gene expression, activating downstream signaling effectors, i.e., calcium/calmodulin-dependent protein kinase II (CaMKII), protein kinase C (PKC), extracellular signal-regulated kinases (ERK), and cAMP response element-binding protein (CREB), which are shared by other different neurotransmission processes [18].

Due to the emerging interest on the effect of thyronamines in brain, the present study sought to explore the impact of T_1_AM and its main catabolite, TA_1_, on signal transduction pathways in brain cell lines. As an in vitro model, we used NG108-15 cells, a hybrid cell line of mouse neuroblastoma and rat glioma, and U-87 MG cells, derived from a human malignant glioma. First, we characterized the experimental models, by assessing uptake and metabolism of T_1_AM and TA_1_; then, we evaluated and compared the effects on proteins involved in signaling cascades, hypothesizing that TA_1_ may strengthen T_1_AM’s actions, especially in brain tissue. We analyzed the effects on cell viability, glucose uptake, second messenger production and expression, and post-translational modifications of proteins involved in signaling cascades after chronic treatment.

## 2. Materials and Methods

### 2.1. Chemicals

T_1_AM was purchased from Cayman Chemical (Ann Arbor, MI, USA). TA_1_ was kindly provided by Dr. Thomas S Scanlan (Oregon Health and Science University, Portland, OR, USA). Unless otherwise specified, all other reagents were from Sigma-Aldrich (St. Louis, MO, USA).

Solvents for HPLC-MS/MS measurements were HPLC grade, and the other chemicals were reagent grade.

The vehicle for T_1_AM and TA_1_ in the cell treatment was dimethyl sulfoxide (DMSO).

A graphical abstract of methods was included in the Supplementary Material (Figure S1).

### 2.2. Cell Treatments

NG108-15 cell line, a hybrid cell line of mouse neuroblastoma and rat glioma, and U-87 MG cell line, from human malignant glioma, were obtained from Sigma-Aldrich (St. Louis, MO, USA).

Cells were cultured in Dulbecco’s Modified Eagle Medium (DMEM), supplemented with 10% (*v*/*v*) of fetal bovine serum (FBS), 1 mM pyruvate, 4.5 g/L glucose, 100 U/mL penicillin, and 100 µg/mL streptomycin at 37 °C in a humidified atmosphere containing 5% CO_2_, and subcultured before confluence.

To assess protein expression, cells were exposed, for 24 h, to exogenous T_1_AM (in a range from 0.1 μM to 10 µM) Control cells were incubated with supplemented DMEM containing DMSO.

Western blotting was performed on the supernatant fraction of cell lysate [19]. The protein concentration was determined by the Bradford method.

### 2.3. Gene Expression Analysis

The expression of eight genes (Glur2, Nmdar1, Nmdar2b, Ephb2, Pkcα, Pkcγ, Sirt1, Erk1) (Tables S1 and S2) was evaluated in NG108-15 and U-87 MG cell lines by real-time PCR, which was performed according to the manufacturer’s instruction (Euroclone, Milan, Italy) in three independent samples.

RNA isolation was performed using chloroform extraction provided as TRIzol Kit (Themo Fisher Scientific, Milan, Italy), and the procedure followed as described in the manufacturer’s protocol.

After resuspended RNA in RNAase free water, all samples were purified performing digestion with DNAase by RNA Clean & Concentrator (Zymo Research, Irvine, CA, USA). RNA concentration and purity were then analyzed using a Qubit RNA HS Assay kit (Life Technologies, Carlsbad, CA, USA) with a Qubit 1.0 fluorometer from Invitrogen (Waltham, MA, USA).

Total RNA was then retrotranscribed (5 min at 25 °C, 20 min at 46 °C, and 1 min at 95 °C) using iScript gDNA Clear cDNA Synthesis Kit (Bio-Rad Laboratories, Hercules, CA, USA).

Relative quantity of gene transcripts was measured by real-time PCR, on samples’ cDNA, using a SYBRGreen chemistry and iQ5 instrument (Bio-Rad). Additionally, 4 µL of 2 µM primer solution were added to 10 µL SsoAdvanced Universal SYBR Green Supermix (Bio-Rad) in a 20 µL total volume reaction. The PCR cycle program consisted of an initial 30 s denaturation at 95 °C, followed by 40 cycles of 10 s denaturation at 95 °C and 15 s annealing/extension at 60 °C. For primers with low Ta, we used a lower temperature for the annealing/extension step. Primers were designed with Beacon Designer Software v.8.20 (Premier Biosoft International, Palo Alto, CA, USA) with a junction primer strategy (Tables S1 and S2 in supplementary material). For the hybrid cell line, we used ClustalW [20] to find the homology region where we designed the primer. In any case, negative control of retro-transcription was performed to exclude any interference from residual genomic DNA contamination.

### 2.4. Uptake of T_1_AM and TA_1_

To evaluate T_1_AM or TA_1_ uptake, experiments were performed, as previously described, with minor modifications [21], and extracts were analyzed by HPLC-coupled to tandem mass spectrometry (HPLC-MS/MS) [21] upon 24 h treatment with T_1_AM or TA_1_, at concentration ranging from 0.1 to 10 µM.

To assess distribution in cellular fractions, cells were treated in flasks with 10 µM T_1_AM for 1 h. The nuclear pellet was extracted by using a nuclear extraction kit (Abcam, Cambridge, UK); the resulting cytoplasmic extract was centrifuged at 10,000× *g*, for 30 min at 4 °C, to separate the mitochondrial and the cytosolic fractions. Fractions were extracted following the same protocol mentioned above.

### 2.5. Cell Viability

To assess cell viability by using the 3-(4,5-dimethylthiazol-2-yl) 2,5-diphenyltetrazolium bromide (MTT) test, cells were treated for 24 h with T_1_AM or TA_1_ at different concentrations (0.1–10 μM), and then, MTT staining was performed [22].

The absorbance of the solution was read at 570 nm in a microplate reader (BioRad Laboratories, Milan, Italy). Results were expressed as a percentage of control.

### 2.6. Glucose Consumption

To assess glucose uptake, cells were exposed, for 4 h, to exogenous T_1_AM or TA_1_ (0.1–10 µM) in DMEM base (phenol red free) supplemented with 0.5 mg/mL glucose [23]. Control group was incubated with DMEM containing the same volume of vehicle. Cell culture medium was then collected, and glucose concentration was evaluated in the medium with a spectrophotometric assay kit (Sigma-Aldrich) at 340 nm. Metabolite concentration referred to the total protein content of whole-cell lysates.

### 2.7. cAMP Concentration Assay

cAMP concentration was assessed in cell lysate with an ELISA assay kit (BioVision Incorporated, Milpitas, CA, USA) according to manufacturer’s instruction. Briefly, cells were treated for 24 h with T_1_AM or TA_1_ (0.1–10 μM). At the end of treatment, 0.1 M HCl was added to stop enzyme activity (phosphodiesterases) The cAMP concentration in supernatant was spectrophotometrically evaluated, and results were normalized to total protein concentration in supernatant.

### 2.8. Protein Expression Analysis

Protein expression analysis was performed according to manufacturer’s instructions (Bio-Rad). In brief, proteins were subjected to SDS-PAGE (4–20% acrylamide separating gel, Criterion stain-free TGX Biorad). The separated proteins were then transferred to a polyvinylidene difluoride (PVDF) membrane (Millipore Corporation, Billerica, MA, USA), which was incubated overnight with the diluted antibody. Primary antibodies against CREB, pCREB, pERK, Sirt1, and c-Fos, and secondary antibodies were purchased from Cell Signaling (Danvers, MA, USA); CAMKII, pCAMKII, ERK, and PKC were purchased from Santa Cruz Biotechnology (Dallas, TX, USA).

Immunoblots were visualized by means of a chemiluminescence reaction (Millipore) by Image LabTM Software (Biorad) under a luminescent image analyzer (Chemidoc XSR+ Bio-Rad). Only bands below the saturation limit were analyzed. The protein level was normalized to the optical density of total proteins in each lane.

### 2.9. Statistical Analysis

Results are expressed as the mean ± SEM. Differences between groups were analyzed by one-way or two-way ANOVA, as detailed for each figure. In the experiments aimed at determining differences vs. a single control group, Dunnett’s post-hoc test was applied. The threshold of statistical significance was set at *p* < 0.05. GraphPad Prism version 6.0 for Windows (GraphPad Software, San Diego, CA, USA) was used for data processing and statistical analysis. 

## 3. Results

### 3.1. Expression of the Receptors of the Glutamatergic Pathway

Both cell lines, NG108-15 and U87MG, were characterized by real time PCR to evaluate the expression of receptors implicated in the glutamatergic postsynaptic pathway. The expression of Nmdar1, Nmdar2b, Glur2, Ephb2, and Taar1 genes was evaluated using a mouse TATA box binding protein (Tbp), as a housekeeping gene for NG108-15 cells and Hypoxanthine phosphoribosyltransferase 1 (Hprt1), for U87 MG cells.

**Figure 1:**
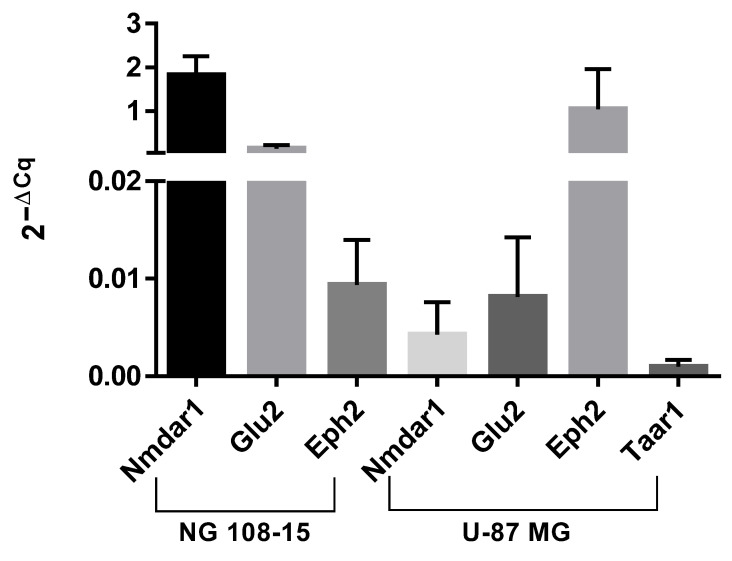
Expression of receptors in NG108-15 and U-87 MG cell lines was assessed by using real time PCR. Values are expressed as mean ± SD of 2^−^^ΔCq^ of three independent samples. Ct of receptors was compared to the housekeeping gene, TATA box binding protein (Tbp), for NG108-15 cells and Hypoxanthine phosphoribosyltransferase 1 (Hprt1) for U87 MG cells.

The receptor expression was evaluated using real-time PCR. As shown in Figure 1, both cell lines expressed receptors, albeit to different extents, if compared to the corresponding housekeeping gene implicated in glutamatergic postsynaptic pathway: Nmdar1, Glur2, and Ephb2. Taar1, the putative T_1_AM receptor, was only expressed in the U87 MG. Other genes, namely Pkcα, Sirt1, and Erk1, whose protein expression was also evaluated, were also expressed; by contrast, neither Pkcγ nor Nmdar2B gene expression was detected in our in vitro models.

### 3.2. Cellular Uptake of T_1_AM and TA_1_

We measured HPLC MS/MS T_1_AM uptake and TA_1_ production in NG108-15 and U-87 MG cell lines, in the presence of FBS and T_1_AM (0.1, 1 or 10 µM), and in cell medium and lysate at the end of treatment (Instrument detection limits: T_1_AM > 0.3 nM; TA_1_ > 5 nM).

As summarized in Table 1, in the medium used to treat NG108-15 cells, after 24 h, T_1_AM was detectable only at the highest concentration of infusion (10 µM) and averaged 0.66 ± 0.14 nM, while its catabolite, TA_1_, was detected starting from the lowest concentration. Similar results were obtained in the U-87 MG cell line, where, in the medium, T_1_AM was present in trace amounts (0.36 ± 0.01 nM, at 10 µM T_1_AM), and TA_1_ was detected at every infused concentration. In cell lysates, T_1_AM was still measurable only at 10 µM T_1_AM and present in trace amounts at the other tested concentrations. Differently, TA_1_ was clearly detectable at either 1 or 10 µM T_1_AM, and it was present in trace amounts at 0.1 µM T_1_AM in both cell lines.

The values of the concentrations of T_1_AM and of its catabolite, TA_1_, in the different cell fractions are summarized in Table 2 and expressed as µM and nM, respectively. The results showed a similar T_1_AM distribution in the two cell lines: T_1_AM was detected in all fractions, albeit at a higher concentration in cytosol and nuclear fractions. Differently, TA_1_ was measurable in all fractions of NG108-15 cells, while in U-87 MG cells, TA_1_ was detected only in cytosol. Due to pellet resuspension, the concentrations of T_1_AM and TA_1_ in the mitochondrial and nuclear fractions might be underestimated. These results indicated a wide distribution of T_1_AM in cells and confirmed that different experimental models may produce diverse behaviors.

To exclude any endogenous production of thyronamines or catabolites, the same experimental procedure was repeated with supplemented DMEM in the absence of exogenous T_1_AM, incubated alone or in the presence of cells: neither T_1_AM nor TA_1_ was revealed.

To complete the uptake, the treatment was repeated with only TA_1_, which was detectable in medium and cell lysate after 24 h of infusion (Table 3). Even though the concentration in the medium was reduced only at 10 µM, TA_1_ was measurable in cell lysates at any tested concentration, indicating that cells could take it up. At 0.1 or 1 µM, we observed values that were higher than the infused concentrations, probably due to a matrix effect, since similar results were obtained with the only medium, after 24 h of infusion, and averaged as follows (nM): 128 ± 13, TA_1_ 0.1 µM; 1102 ± 42, TA_1_ 1 µM; 10,392 ± 200, TA_1_ 10 µM. No further metabolism of TA_1_ was recorded during infusion. T_1_AM was present in trace, maybe as an impurity of TA_1_, since neither T_1_AM nor TA_1_ were revealed in a previous assessment of the experimental model.

### 3.3. Cell Viability

**Figure 2:**
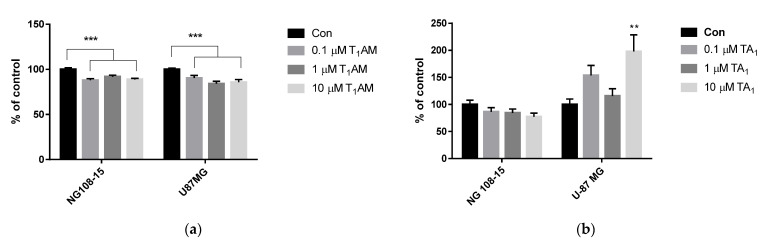
Cell viability of NG108-15 or U-87 MG cell lines, after 24 h treatment (**a**) with T_1_AM and (**b**) with TA_1_, by using MTT test. All treatments received the same amount of vehicle. Control groups (Con) were incubated with medium containing only vehicle (DMSO). Data are plotted as means ± SEM and expressed as % of control [two-way ANOVA, *p* < 0.01, Dunnett’s post-hoc test for multiple comparison, ** *p* < 0.01, *** *p* < 0.001 vs. Control.

Cell viability was evaluated by MTT test in NG108-15 and U87 MG cells (Figure 2) treated with different concentrations of T_1_AM or TA_1_.

In both cell lines, T_1_AM showed a slight—but significant—cytotoxic action starting from 0.1 μM (Figure 2a, −10–15%, *p* < 0.001 vs. control), implying a decrease oxidative metabolism. Below this concentration, T_1_AM was not cytotoxic (0.01 µM vs. control: NG108-15 cells, 101 ± 1.7 vs. 100.0 ± 1.7; U-87 MG cells, 96.1 ± 1.7 vs. 100.0 ± 1.5; *p* = NS).

TA_1_ was not cytotoxic, and a significant increase, by 50%, was measured after treatment with TA_1_ at 10 µM (** *p* < 0.01) in the U-87 MG cell line, while no change was observed in the NG 108-15 cell line (Figure 2b).

### 3.4. Glucose Consumption

To assess glucose consumption, NG 108-15 and U-87 MG cells were incubated for 4 h in phenol red-free DMEM containing 0.5 g/L glucose. At the end of treatment, glucose concentration was assayed in the medium, and the results were expressed as the difference between the initial and final concentrations normalized to the total protein content in cell lysates.

**Figure 3:**
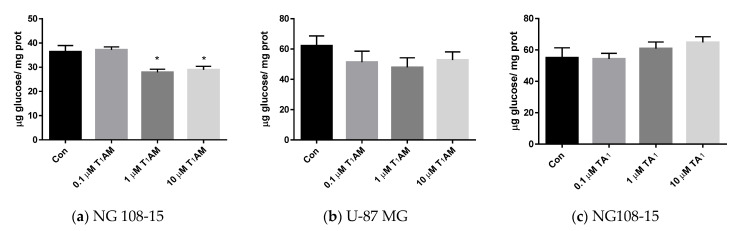
Glucose consumption was evaluated using a spectrophotometric assay kit, after 4 h of treatment: (**a**) with T_1_AM in NG108-15 cells, (**b**) with T_1_AM in U87-MG cells, and (**c**) with TA_1_ in NG108-15 cells. Results are the difference between the initial glucose concentration in medium and the final concentration, normalized to the total content of proteins in cell lysate. Control cells (Con) were incubated with a medium containing the same volume of vehicle. Values are mean ± SEM and are expressed as µg glucose/mg of total protein in lysate. [(**a**) T_1_AM, one-way ANOVA, *p* < 0.05, Dunnett’s post-hoc test for multiple comparison, * *p* < 0.05, vs. control (Con); n = 4 per groups].

As indicated in Figure 3a, a 20% decrease in glucose consumption was observed upon 4 h treatment at 1–10 µM T_1_AM in the NG108-15 cell line (*p* < 0.05 vs. control by one-way ANOVA test and Dunnett’s test), while no significant change was observed after treatment in the U-87 MG cell line (Figure 3b *p* = NS vs. control). By comparison, glucose consumption was not affected in NG 108-15 cells exposed to TA_1_ (Figure 3c)

### 3.5. cAMP Assay

The concentration of cAMP was evaluated using a colorimetric assay kit, as described above, after 24 h of treatment with T_1_AM or TA_1_ at concentration, ranging from 0.1 µM to 10 µM.

**Figure 4:**
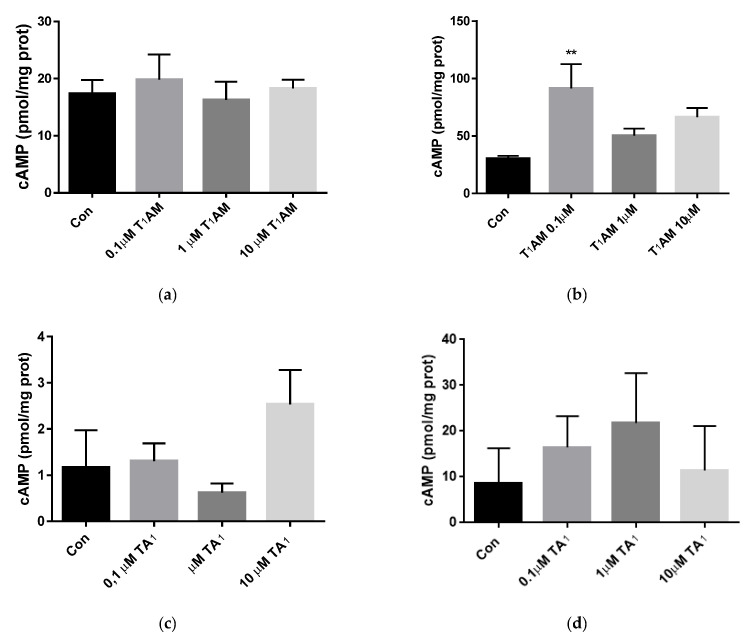
cAMP production was evaluated using a spectrophotometric assay kit, after 24 h of treatment (**a**) with T_1_AM in NG108-15 cells, (**b**) with T_1_AM in U-87 MG cells, (**c**) with TA_1_ in NG108-15 cells, (**d**) with TA_1_ in in U-87 MG cells, ranging 0.1–10 µM. cAMP concentration in each sample was normalized to the total content of proteins in cell lysate. Control cells (Con) were incubated with a medium containing the same amount of vehicle. Values are mean ± SEM and are plotted as pmol/mg of protein. [(**b**), T_1_AM, one-way ANOVA, *p* < 0.05, Dunnett’s post-hoc test for multiple comparison, ** *p* < 0.01, vs. control (Con)].

As shown in Figure 4, only 0.1 µM T_1_AM increased cAMP production in the U87-MG cell line (Figure 4b, *p* < 0.01 vs. Con). No further change was observed in the other treatments with T_1_AM or TA_1_.

### 3.6. Effects of T_1_AM and TA_1_ on the Expression of Signaling Cascade

We investigated changes in expression and post-translational modification of some proteins involved in the glutamatergic postsynaptic signaling, in NG 108-15 and U-87 MG cells, upon infusion of T_1_AM or TA_1_. Protein expression analysis was performed after 24 h of treatment with T_1_AM or TA_1_, ranging from 0.1 to 10 M, resulting in different effects according to the cell line.

The major effect highlighted in both cell lines was an increase in the phosphorylation of members of the signaling cascade, as shown in Figure 4 (and in Supplementary Material, Figure S2), albeit on different target proteins.

**Figure 5:**
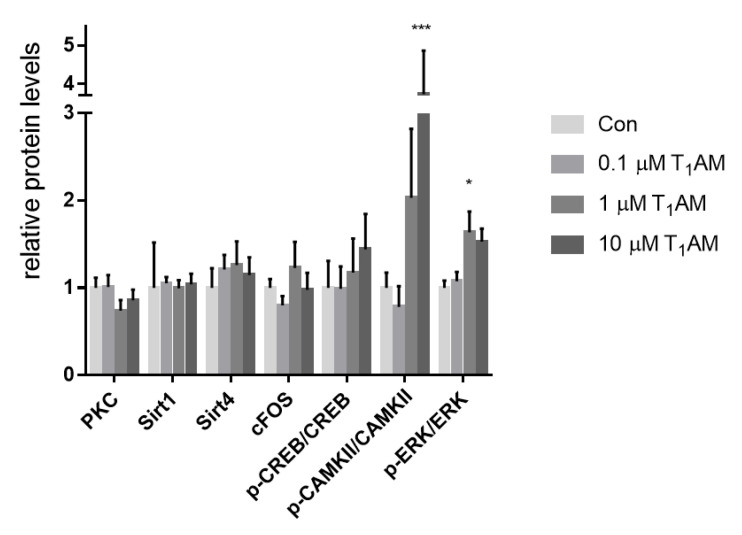

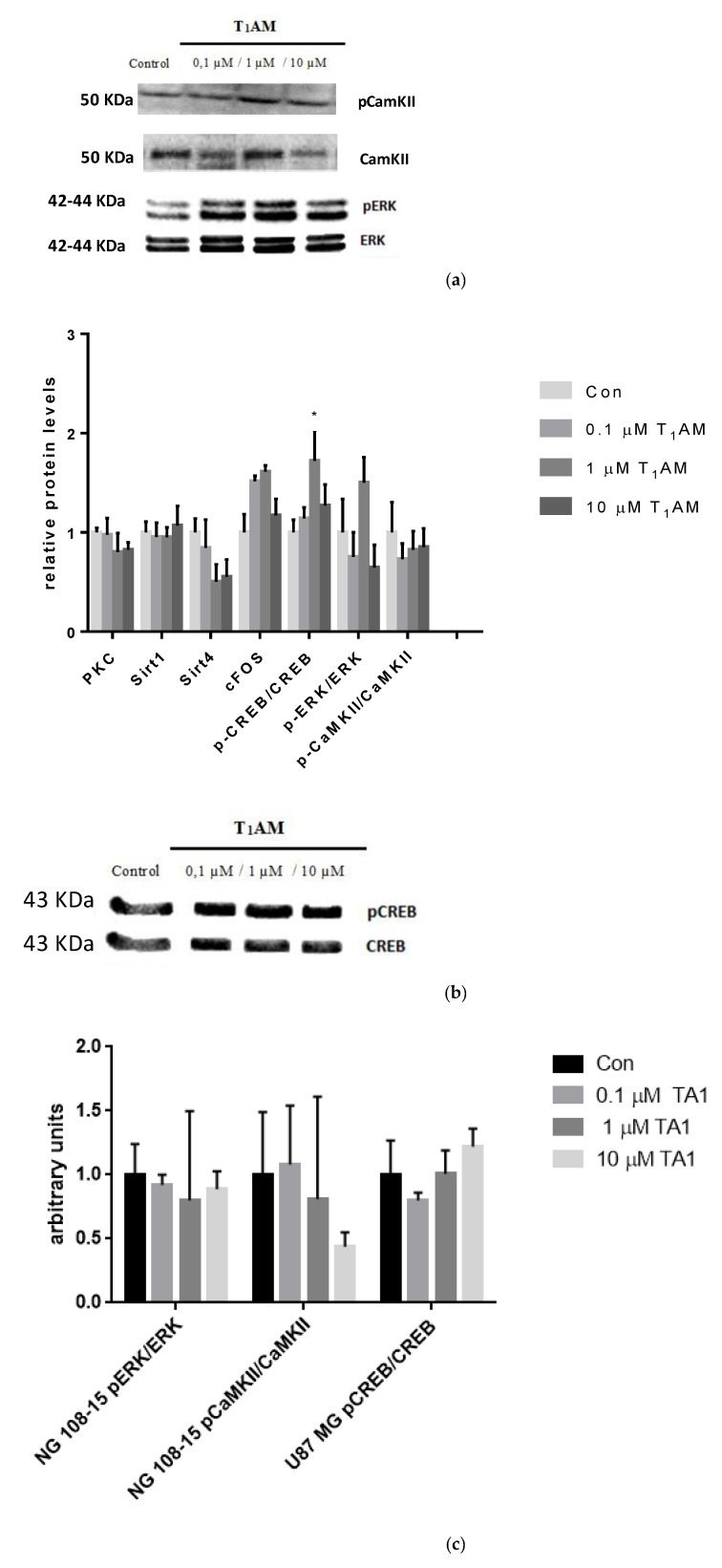
Western blot analysis of T_1_AM or TA_1_ effects on protein expression, belonging to the glutamatergic system, after 24 h treatment. (**a**) T_1_AM in NG108-15 cells, (**b**) T_1_AM in U-87 MG cell lines, and (**c**) TA_1_ in NG108-15 cells. Histograms represent mean ± SEM. Representative immunoblots of proteins, which reached significance, are shown. All results are normalized against total proteins, measured using stain-free gels, and are expressed as relative protein levels. All treatments received the same amount of vehicle, and control group (Con) was incubated with a medium containing only vehicle (DMSO). Two way ANOVA, *p* < 0.001, and Dunnett’s post-hoc test are used for multiple comparisons, * *p* < 0.05, *** *p* < 0.001 vs. control, Con (**a**,**b**)].

In the NG 108-15, an increase in phosphorylation of ERK and CaMKII was observed at the highest T_1_AM concentrations (Figure 5a, 1 µM T_1_AM, pERK/ERK + 60%, * *p* < 0.05 vs. Control; Figure 5a, 10 µM T1AM, pCAMKII/CAMKII + 50%, * *p* < 0.05 vs. control), while in U87 MG cells, T_1_AM induced the phosphorylation of the transcriptional factor CREB at 1 µM (Figure 5b, pCREB/CREB, + 70%, *p* < 0.01 vs. control). Neither expression nor post-translational modifications of other proteins were affected. We also investigated changes by TA_1_ in expression and post-translational modifications in proteins, which were significantly affected by T_1_AM (Figure 5c): neither in U-87 MG, nor in NG 108-15 cells, the phosphorylation of the above-mentioned proteins was altered upon infusion of TA_1_, indicating that effects may be attributed to T_1_AM, rather than to the production of its catabolite TA_1_.

## 4. Discussion

In this project, we firstly characterized the experimental model, and then, we evaluated the effects of T_1_AM on proteins involved in signal transduction pathways. Since T_1_AM was metabolized to TA_1_, experiments with significant results were extended to TA_1_ with the aim of evaluating if its catabolite might reproduce T_1_AM’s effects.

Our results indicated that NG108-15 and U-87 MG cell lines expressed, although to a different extent, the main receptors of the glutamatergic system: namely, Nmdar1, Glur2, Ephb2, but not Nmdar2b. Taar1, the putative T_1_AM receptor, was expressed only by U87 MG cells, making the NG108-15 cell line a valid negative control for effects that are assumed to be mediated by this receptor.

NMDA receptors consist in four subunits: two obligatory NR1 subunits and two regulatory subunits that can be NR2A→D, or NR3A→B. The precise combination of NMDAR subunits determines the functional properties of the NMDAR channels [24]. NR2A and NR2B subunits, which are the predominant NMDAR subtypes in the forebrain, undergo a particularly well-characterized developmental shift in the cortex. NR2B subunits are abundant in the early postnatal brain, and NR2A levels increase progressively with development [25,26,27]. The absence of Nmdar2b may be justified by the fact that the NR2B subunit is usually expressed only in some regions of brain and only in the postnatal stage, while subunit NR1 is widely expressed in adult human and mouse brain but only in the embryo rat brain [28]. EphB-ephrinB, particularly B2, interaction has recently emerged as major role player in synaptic plasticity and neuronal process development, including long term potentiation [29]. EphrinB2 signaling is critical for the stabilization of AMPA receptors at the cellular membrane, and its expression is elevated in cancer cell lines, such as neuroblastoma and glioma, compared to primary cell lines [30]. Both cell lines expressed Pkcα, Erk1, and Sirt1 proteins involved in several post synaptic signaling cascades, while Pkcγ was not detected. Protein kinases PKCα and PKCγ belong to the family of PKC activated by cytosolic Ca2+ ions and diacylglycerol; while the alpha isoform is expressed in several brain regions and other tissues, only brain, spinal cord, cerebellum, hippocampus, and cerebral cortex have been found to express the Pkcγ isoform [31]. The activity of the Pkcα contributes to the enhancement of plasticity in the hippocampal CA1 region. Indeed, the inhibition of this kinase can eliminate long term potentiation [32].

To complete the validation of our models, we assessed the uptake of exogenous T_1_AM and TA_1_ in the cell medium and lysate at the end of treatment (24 h) and their metabolism. We observed that T_1_AM, incubated in standard cell culture medium supplemented with FBS, was absorbed by U87 MG and NG108-15 cell lines, diffused into the cell fractions, and rapidly catabolized to TA_1_, which was measurable in all fractions of NG 108-15 cells, while in U-87 MG cells, it was detected only in cytosol. These results indicated a wide distribution of T_1_AM into the cell and confirmed that different experimental models may produce diverse experimental response. No further metabolism was observed with exogenous TA_1_.

The last step of cell characterization was to test T_1_AM cytotoxicity. T_1_AM showed a slight cytotoxic effect in both NG108-15 and U87 MG cells, as revealed from the MTT test, also implying a reduced oxidative metabolism. Differently, TA_1_ did not affect cell viability: this could be explained by considering that no further oxidative deamination occurred in presence of TA_1_, and consequently, there was no production of hydrogen peroxide, ammonia, or other compounds, which are usually produced by monoamino oxidase catalysis and can alter the redox state of cells and induce cytotoxicity [33].

Thus, both NG108-15 and U87 MG cell lines expressed proteins associated with the glutamatergic and other systems of signal transduction pathways, and they may be considered an in vitro model to evaluate effects of T_1_AM or TA_1_ on different intracellular cascades.

We then investigated changes in expression and post-translational modifications of some of these proteins upon infusion of T_1_AM. The major effect we observed was an increase in phosphorylation, exerted by T_1_AM, but not reproduced by TA_1_ alone. We showed that T_1_AM was able to induce phosphorylation of nuclear factor CREB in U-87 MG cells, while it was able to induce phosphorylation of CaMKII and ERK in NG108-15 cells. These findings indicated that infusion with exogenous T_1_AM might alter the glutamatergic signaling cascades and, in general, other signal pathways which contain this kind of protein kinases, albeit with different effects, depending on the in vitro model used.

Both cell lines shared a similar receptor pattern, except for TAAR1, which was not expressed in NG108-15 line: this might account for differences in sensitivity towards exogenous T_1_AM. Notably, most significant effects were recorded at T_1_AM 10 µM, the only treatment which allowed the detection of T_1_AM in the medium after 24 h, indicating that effects could be attributable to T_1_AM rather than to its catabolite, as confirmed by treatment with TA_1_.

CaMKII is a kinase activated by calcium ions, a key mediator in connecting transient calcium influx to neuronal plasticity, and its autophosphorylation at Thr-286 induces a persistent activation [34]. This kinase phosphorylates and activates different substrates, including the extracellular regulated kinase (ERK), which, in turn, leads to the activation of transcription factors, CREB and c-fos among them, and then, the gene transcription that triggers the synthesis of new proteins. The transcriptional factor CREB is the heart of the glutamatergic signaling cascade, and its activation at the end of the cascade leads to the transcription of factors fundamental for memory consolidation and LTP [35]. We have to take into account that ERK enzymes are points of convergence for several pathways, and the wide range of effects exerted by ERK activation in downstream cascades of different signaling system has been well established [36].

The observed increase in phosphorylation may underlie the potential mechanism of T_1_AM of its prolearning effects. Even though our hypothesis is not fully sustained by the increase in the second messenger cAMP, which was almost unchanged, we can hypothesize that a cross talk between pathways might have occurred. Moreover, to confirm this hypothesis, it could be of interest to evaluate changes in protein phosphorylation at different time course points.

Glucose is the main energetic substrate for the brain. Differences between cell lines were encountered in glucose consumption as well, which was slightly decreased after 4 h by 1–10 μM T_1_AM, only in the NG108-15 cells, in contrast with the need of ATP to support protein phosphorylation. Assadi-Porter et al. [37] demonstrate that T_1_AM can act as a regulator of both glucose and lipid metabolism in mice through sirtuin-mediated pathways, but in our model, no change in sirtuins was observed.

Our biochemical observations are partially consistent with previous results [10,37]: differences may be attributed to different circumstances, such as environmental conditions, cell stages/passages, subcellular localization, experimental models, and procedures.

However, our study suffers from limitations in experimental models. These cell lines are an unlimited auto-replicative source, relatively easy to culture, but they suffer from the limitations induced by a cancer cell, which expresses a particular gene profile. For this reason, the assessment of the overall effects of T_1_AM and TA_1_ should be extended to primary cell lines (astrocytes or primary neuronal/hippocampal cells) and, then, to in vivo experimental models, which are more relevant and reflective of the original environment.

## 5. Conclusions

The cell lines investigated express receptors and signaling molecules implicated in several signaling pathways, and they can uptake and metabolize T_1_AM.

Most of the experiment results are statistically significant only at the highest concentration of T_1_AM. The observed effects are mediated, mainly, by T_1_AM rather than its metabolite, TA_1_; as a matter of fact, all the significant changes by T_1_AM were not reproduced by the infusion of TA_1_ alone. This may indicate that the effects induced by T_1_AM were not influenced or strengthened by the production of TA_1_, as described differently in the literature on animal models [33].

T_1_AM can affect biological processes mediated by phosphorylation of proteins in several pathways, including the glutamatergic system. It demonstrates a very complex pharmacology, and an alteration in these signaling molecules or phosphorylation could not be its only primary effect, but other mechanisms may also be implicated. These findings should be considered during the development of potential T_1_AM-based therapies.

## Figures and Tables

**Table 1:** Concentrations of T_1_AM and TA_1_ in medium and cell lysate after 24 h of infusion. Data represent mean ± SEM, n = 3–4 per group, and are expressed as nM. T_1_AM or TA_1_ contents were measured in medium and lysate of NG108-15 or U-87 MG cells, which were incubated for 24 h with T1AM (1–10 µM). [*p* < 0.0001 for TA1 in medium and lysate in both cell lines (ANOVA)]. N.D., Not Detectable.

T1AM	NG 108-15 Cells				U-87 MG Cells			
	Medium		Cell Lysate		Medium		Cell Lysate	
(µM)	T_1_AM (nM)	TA_1_ (nM)	T_1_AM (nM)	TA_1_ (nM)	T_1_AM (nM)	TA_1_ (nM)	T_1_AM (nM)	TA_1_ (nM)
0.1	N.D.	135 ± 12	N.D.	N.D.	N.D.	95 ± 6	N.D.	N.D.
1	N.D.	311 ± 161	N.D.	7.7 ± 0.2	N.D.	1165 ± 41	N.D.	22 ± 5
10	0.66 ± 0.14	4996 ± 97	10 ± 6	91 ± 19	0.36 ± 0.01	13,214 ± 302	6 ± 0.3	144 ± 80

**Table 2:** Concentrations of T_1_AM and TA_1_ in cellular fractions after 1 h of infusion. Data represent mean ± SEM, n = 3 per group, and are expressed as µM (T_1_AM) or nM (TA_1_). T1AM or TA1 contents were measured in mitochondrial, nuclear, and cytosolic fractions of NG108-15 or U-87 MG cells that were incubated for 1 h with 10 µM T_1_AM. [Within each row, *p* < 0.0001 for T_1_AM, and *p* < 0.001 for TA_1_ for differences among cellular fractions (ANOVA)]. N.D., Not Detectable.

Cell Lines	Cytosolic Fraction		Mitochondrial Fraction		Nuclear Fraction	
	T_1_AM (µM)	TA_1_ (nM)	T_1_AM (µM)	TA_1_ (nM)	T_1_AM (µM)	TA_1_ (nM)
NG108-15	2.89 ± 0.13	205.3 ± 27.6	1.66 ± 0.1	18.6 ± 2.9	2.77 ± 0.07	46.5 ± 9.3
U-87 MG	2.62 ± 0.24	20.5 ± 0.9	0.54 ± 0.17	N.D.	1.63 ± 0.34	N.D.

**Table 3:** Concentrations of TA_1_ in medium and cell lysate after 24 h of infusion. Data represent mean ± SEM, n = 3–4 per group and are expressed as nM. TA_1_ content was measured in medium and lysate of NG108-15 or U-87 MG cells, which were incubated for 24 h with TA_1_ (1–10 µM). [*p* < 0.0001 for TA_1_ in medium and lysate in both cell lines (ANOVA)].

Cell Lines/TA_1_	Medium (nM)			Lysate (nM)		
	0.1 µM	1 µM	10 µM	0.1 µM	1 µM	10 µM
NG 108-15	233 ± 18	1795 ± 55	5355 ± 175	6.8 ± 1.9	72.4 ± 6.9	995 ± 95
U-87 MG	135 ± 26	1193 ± 140	1520 ± 327	1.6 ± 0.1	15.4 ± 3.9	120 ± 22

## Data Availability

Raw data of this research study are available by contacting the authors.

## Supplementary Materials

The following are available online at https://www.mdpi.com/article/10.3390/life12091352/s1, Figure S1: Graphical abstract showing all methods used in our study, Figure S2: Western blotting: Representative immunoblots of proteins after 24 h of treatment with T_1_AM in NG108-15(A) and U-87 MG (B) cell lines, or with TA_1_ (C), Table S1: Primer sequences of target genes used in hybrid cell line NG108-15. Homology gene sequences of rat and mouse were found using ClustalW [20] and primers were designed with Beacon Designer Software v.8.20 on these regions, Table S2: Primer sequences of target genes used in the human cell line U-87 MG. Primers were designed with Beacon Designer Software v.8.20.
