# Supplementary material for: Exogenous 3-Iodothyronamine (T1AM) Can Affect Phosphorylation of Proteins Involved on Signal Transduction Pathways in In Vitro Models of Brain Cell Lines, but These Effects Are Not Strengthened by Its Catabolite, 3-Iodothyroacetic Acid (TA1)
Source: Life (Basel). 2022 Aug 30;12(9):1352. doi: 10.3390/life12091352 (PMC9502970; doi:10.3390/life12091352)
Supplement: Supplementary file 1 [file life-12-01352-s001.zip › life-1839364-supplementary.pdf]

**Supplementary material**

**Figure S1**

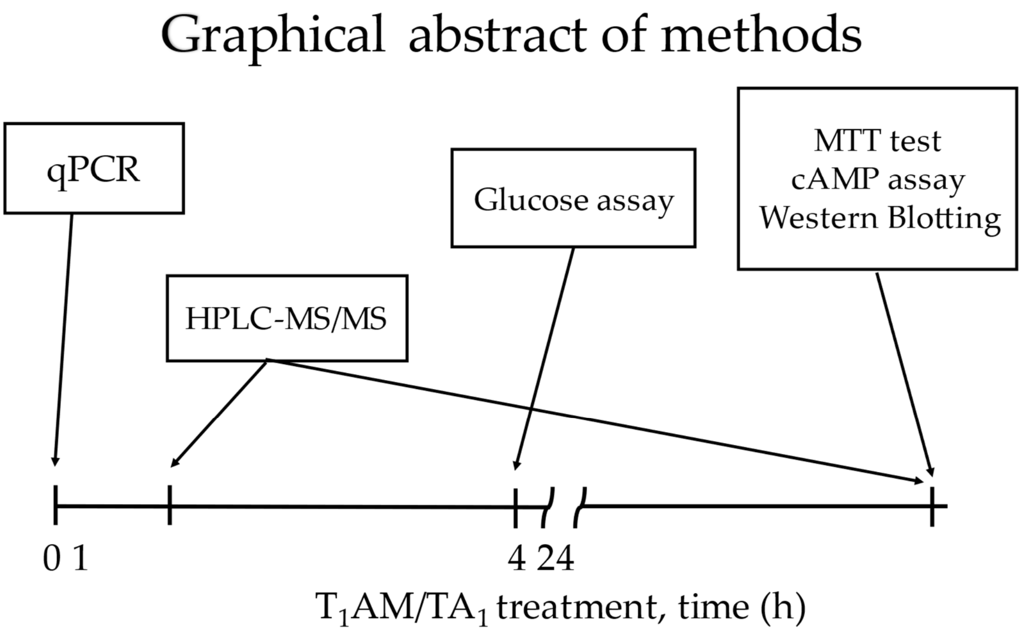


**Figure S1**. Graphical abstract showing all methods used in our study.

**Table S1**

| Gene Symbol | Ta (°C) | Accession nr. | Forward Primer | Reverse Primer |
|---|---|---|---|---|
| Nmdar1 | 60 | NM_008169.3<br>NM_01287423.1 | AATTCAATGAGGATGGGGAC | CATTTGGGATGACATGGGTA |
| Nmdar2B | 60 | NM_012574.1<br>NM_008171.4 | TGTGCCAGGTCGTTTCCA | CCTCACTCAGACATCAGACTCAA |
| Glur2 | 60 | NM_013540.3<br>NM_001083811.1 | TTCTGTCCTCCTTTCTCCT | CGAAACTGTTGGCTACCT |
| EphB2 | 53 | NM_001127319.1<br>NM_001290753.2 | TATCTCAGATGATGATGGA | TGGATACTGTTCAGGATT |
| Taar1 | 60 | NM_134328.1 | CAAGTTGGATTGGAAGGGGAAAGC | TCAGAGTGGGTGGGATAACATAGC |
| Sirt1 | 53 | NM_001159589.2<br>XM_008772947.2 | TCTCTGTGTCACAAATTCATA | CCTGCTCCAAGGTATCTA |
| Erk1 | 60 | NM_017347.2<br>NM_011952.2 | CGGATTGCTGACCCTGAG | ATTTGGTGTAGCCCTTGGA |
| Pkcα | 60 | NM_011101.3<br>NM_001105713.1 | GCCACAAGCAGTATTCTA | CTGGTAAGCGATTATCTCT |
| Pkcγ | 60 | NM_012628.1<br>NM_011102.4 | GATAGGACATCCCGAAAT | CGCACTCTCTCATACAAT |
| Tbp | 60 | NM_013684 | GCCTTCCACCTTATGCTC | AGTAAGTCCTGTGCCGTAA |

**Table S1**: Primer sequences of target genes used in hybrid cell line NG108-15. Homology gene sequences of rat and mouse were found using ClustalW [20] and primers were designed with Beacon Designer Software v.8.20 on these regions.

**Table S2**

| Gene Symbol | Ta (°C) | Accession nr. | Foward Primer | Reverse Primer |
|---|---|---|---|---|
| Nmdar1 | 60 | NM_001185091.1 | CAAGTGGGCATCTACAAT | CGTCACAATCTTCAGTCT |
| Nmdar2B | 60 | NM_000834.4 | CCTGGAATGGTATGATTG | CTGAATGGCTCTAAGAAG |
| Glur2 | 60 | NM_001083620.1 | CGTCTCCTTCATCACTCC | GATAAGCCTCTGTCACTGT |
| EphB2 | 60 | NM_001309193.1 | AACCAGGATGTAATCAATG | ATCTTGTCTAGCGTGTTG |
| Taar1 | 60 | NM_138327 | ACTTTCTTCTGGGGTGTCTGGTC | CACAGCATAGTAGCGGTCAATGG |
| Sirt1 | 60 | NM_001314049.1 | GTAGGCGGCTTGATGGTAAT | GGGTTCTTCTAAACTTGGACTCT |
| Pkcγ | 60 | NM_002739.4 | TGGATGGCTGGTACAAGTTA | CGCACCCGCTCATACAAT |
| Hprt1 | 60 | NM_000194 | ATACAAAGCCTAAGATGAGAGTTC | AAACAACAATCCGCCCAAAG |

**Table S2**: Primer sequences of target genes used in the human cell line U-87 MG. Primers were designed with Beacon Designer Software v.8.20.

**Figure S2**

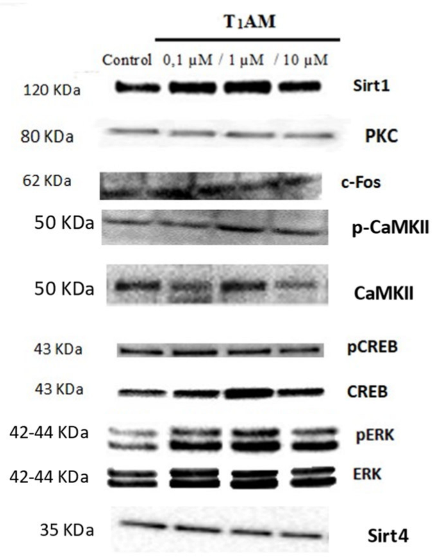


(A) Representative blots of proteins in NG108-15 cell line.

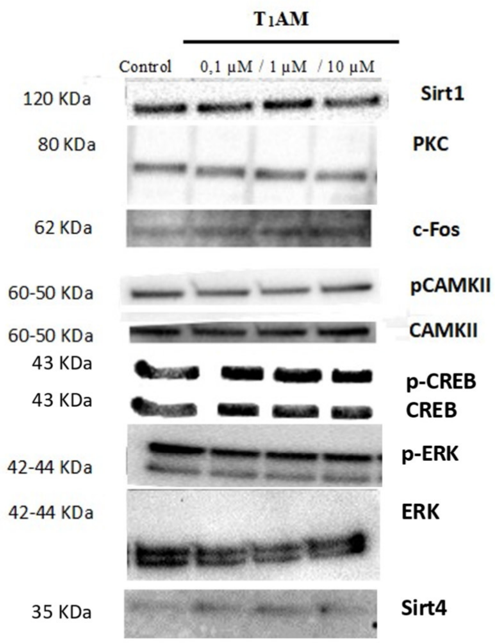


(B) Representative blots of protein in U87-MG cell line

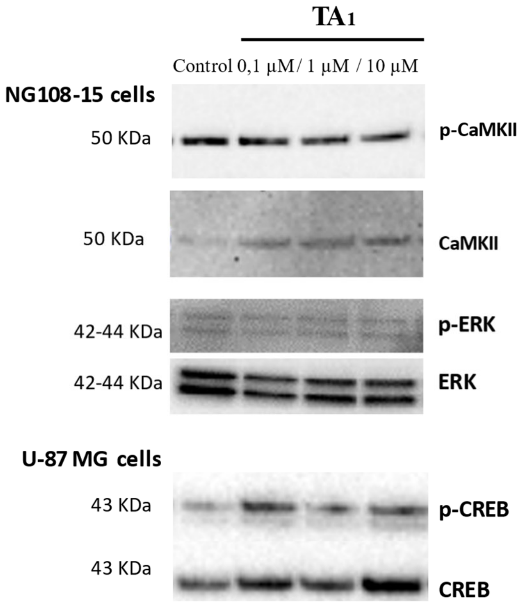


(C) Representative blots of protein in U87-MG or in NG108-15 cells treated with $TA_1$

**Figure S2**. Western blotting: Representative immunoblots of proteins after 24h of treatment with $T_1AM$ in NG108-15 (A) and U-87 MG (B) cell lines, or with $TA_1$ (C).
